# The Italian Version of the ALS Depression Inventory-12

**DOI:** 10.3389/fneur.2021.723776

**Published:** 2021-09-24

**Authors:** Debora Pain, Edoardo Nicolò Aiello, Marcello Gallucci, Massimo Miglioretti, Gabriele Mora

**Affiliations:** ^1^Istituti Clinici Scientifici Maugeri, IRCCS, Neurorehabilitation Department of Milano Institute, Milan, Italy; ^2^School of Medicine and Surgery, University of Milano-Bicocca, Monza, Italy; ^3^PhD Program in Neuroscience, University of Milano-Bicocca, Monza, Italy; ^4^Department of Psychology, University of Milano-Bicocca, Milan, Italy

**Keywords:** amyotrophic lateral sclerosis, depression, psychometrics, apathy, neuropsychology, frontotemporal dementia

## Abstract

**Introduction:** Depression is a comorbidity in patients with amyotrophic lateral sclerosis (ALS). However, its diagnosis is challenged by the co-occurrence of a similar frontotemporal (FT) behavioral symptom—i.e., apathy. Moreover, its psychometric evaluation is confounded by motor disabilities. This study aimed at investigating psychometric properties and feasibility of the ALS Depression Inventory (ADI-12), a self-report questionnaire set up for this issue—as measuring mood changes without referring to movement.

**Methods:** Eighty-five ALS patients were administered the ADI-12 and underwent cognitive (Mini-Mental State Examination, MMSE), quality of life (McGill Quality of Life Questionnaire, MQoL) and further anxiety/mood (Hospital Anxiety and Depression Scale, HADS) assessments. Reliability, validity, sensitivity, and specificity of the ADI-12 were explored.

**Results:** Principal component analyses revealed two related components—“Negative Mood and Lack of Energy” (ME) and “Anhedonia” (A). Both components and the inventory as a whole were internally consistent and highly related to HADS-D. ADI-12-total score was also associated with HADS-A. ADI-12 measures were inversely related to MQoL. ADI-12-total/sub-scales were not related to either MMSE or disease-related outcomes. Estimates of depression yielded by HADS-D and ADI-12 were 11.1 and 35.3%.

**Discussion:** The ADI-12 is a valid, reliable and usable feasibile tool to assess depression in Italian ALS patients independently from motor disabilities. Its interplay with psycho-social outcomes is in agreement with previous studies. The lack of association with cognition suggests that the ADI-12 is partially independent from FT spectrum disorders. The disagreement in depression rates between the ADI-12 and HADS-D suggests the need to ALS-specific mood scales.

## Introduction

Depression is a well-known psychiatric co-morbidity in patients affected by amyotrophic lateral sclerosis (ALS) (1, 2)—although its etiology still needs to be clarified (3). A diagnosis of depression in ALS population may be confounded by the presence of concomitant apathy (3)—a frontotemporal (FT) behavioral symptom involving up to 50% of ALS patients (4).

Screening for depressive symptoms should not be neglected in ALS patients, as negatively influencing disease prognosis and survival (5). However, psychometric evaluation of mood in ALS patients is challenging as widely used scales to assess depression include items that often refer to motor abilities (6). To adopt assessments not depending on motor impairment is crucial, as its confounding effect has been linked to the heterogeneity in estimating the prevalence of depression in this population (1). Nonetheless, according to recent meta-analytic evidence (7), depressive disorders are estimated to be moderately prevalent in ALS patients (34%).

The ALS Depression Inventory-12 (ADI-12) (8, 9) is an ALS-specific, 12-item, self-report questionnaire whose items measure mood changes without referring to motor function.

This study aimed at investigating psychometric properties and feasibility of the ADI-12 in a cohort of Italian ALS patients.

## Methods

### Participants

Eighty-five ALS patients referred to Istituti Clinici Scientifici Maugeri, IRCCS Milano, Italy, were consecutively recruited. Exclusion criteria were major internal, neurologic or (other) psychiatric disorders. Participants' background and clinical features are shown in Table 1. The study was approved by the local Ethical Committee. Participants provided written informed consent to participation.

**Table 1:** Patients' background and clinical data.

*N*	85	
**Age (years)**	**62.3 ± 9.5 (37-79)**	
**Sex (M/F)**	**51.8/48.2%**	
**Education**	**Elementary School**	**25.9%**
	**Junior high School**	**41.2%**
	**High school**	**25.9%**
	**University**	**7.1%**
**Occupation**	**Retired**	**58.8%**
	**Unoccupied**	**2.4%**
	**Occupied**	**38.8%**
**Age at onset (years)**	**59.7 ± 9.6 (35-77)**	
**Age at diagnosis (years)**	**60.7 ± 9.6 (35-77)**	
**Diagnostic delay (years)**	**0.99 ± 0.63 (0-4)**	
**Disease duration (years)**	**1.6 ± 1.73 (0-13)**	
**Site of onset**	**Bulbar**	**20%**
	**Spinal**	**80%**
Phenotype^*^	**Classical**	**76.2%**
	**Bulbar**	**16.7%**
	**Pyramidal**	**3.6%**
	**FAS**	**2.4%**
	**FLS**	**1.2%**
**MMSE**	**27.61 ± 2.1 (23-30)**	
**ADI-12**	**25.56 ± 5.61 (13-43)**	
**HADS-A**	**6.59 ± 3.67 (1-17)**	
**HADS-D**	**5.14 ± 3.14 (0-14)**	
**MQoL**	**Total**	**6.8 ± 1.25 (3.54-9.5)**
	**Physical**	**5.81 ± 2.27 (1.25-10)**
	**Psychological**	**5.03 ± 1.95 (0.83-9.5)**
	**Existential**	**7.21 ± 1.64 (0-10)**
	**Support**	**8.11 ± 1.59 (0-10)**

*FAS, flail arm syndrome; FLS, flail leg syndrome; MMSE, Mini-Mental State Examination; ADI-12, ALS Depression Inventory-12; HADS-A/-D, Hospital Anxiety and Depression Scale-Anxiety/-Depression; MQoL, McGill Quality of Life Questionnaire*.

* *Piemonte and Valle d'Aosta Register for ALS (PARALS) (10)*.

### Materials

Items were back-translated by two independent Authors (D.P. and M.M.). Inter-rater agreement was assessed and discrepancies were solved through discussion. ADI-12 items are reported through a four-point Likert scale; items 5-9, 11, and 12 are negatively worded.

The protocol for the Italian ADI-12 will be provided to interested clinicians upon request to the corresponding Author.

In addition, patients were tested for cognition (Mini-Mental State Examination; MMSE) (11–13), quality of life (McGill Quality of Life Questionnaire; MQoL) (14), and mood/anxiety (Hospital Anxiety and Depression Scale; HADS) (15). HADS items referring to movement were discussed with patients during administration in order to avoid biases in responses; a similar approach has been adopted (deleting those motor-related items) (16).

### Statistical Analyses

SPSS 27 (17) was used to analyze data.

Raw variables were descriptively checked for normality to determine whether to adopt linear models or non-parametric alternatives (18).

Factorial structure was assessed by means of principal component analyses (PCA). Convergent validity was tested by means of parametric/non-parametric correlational techniques. Reliability was assessed as internal consistency through Cronbach's α.

The optimal cut-off for the ADI-12 was identified through receiver-operating curve (ROC) analyses by computing Youden index (19).

## Results

An obliquely rotated PCA (Table 2) yielded two correlated components (*r* = 0.48) cumulatively explaining 61.02% of variance—the first identified as “Negative Mood and Lack of Energy” (ADI-12-ME; *N* = 7 items), whereas the second as “Anhedonia” (ADI-12-A; *N* = 5 items) (9). Items of the two components can be summed up to obtain the 12-item inventory. No clearly ambiguous items were detected when inspecting the pattern matrix (primary loadings ≥ |0.3|), with the exception of item 10, whose ratio between its primary (*r* = −47) and secondary (*r* = −0.26) loadings was slightly lower than 2 (1.81).

**Table 2:** Loadings of ADI-12 items on the two components as yielded by PCA.

		**Component**	
**Item**		**ME**	**A**
1	I am happy and I smile often.	–**0.86**	0.10
2	I can appreciate life despite my circumstances.	−0.29	–**0.63**
3	I can get away from it all and I am often relaxed.	–**0.85**	0.18
4	I feel alive and vital.	–**0.63**	−0.24
5	More often than not I am sad.	**0.73**	0.1
6	I have lost all interest in family and friends.	−0.14	**0.89**
7	Most often I feel empty.	−0.02	**0.78**
8	There is nothing that I look forward to or that I can enjoy.	0.06	**0.84**
9	I often feel lost and abandoned and don't know how to carry on.	**0.81**	0.02
10	I look forward to every new day.	–**0.47**	−0.26
11	I often wish I were dead.	0.22	**0.58**
12	I feel like I have lost all of my energy.	**0.64**	0.2

*ADI-12, ALS Depression Inventory-12; ME, Negative Mood and Lack of Energy component of ADI-12; A, Anhedonia component of ADI-12; PCA, principal component analysis. They are the acronyms for the psychometric measures*.

According to the aforementioned sub-division, both ADI-12-ME and ADI-12-A items proved to be highly internally consistent (Cronbach's α = 0.86 and = 0.84, respectively), as well as the inventory as a whole (Cronbach's α = 0.9).

All ADI-12 measures displayed medium-to-large correlation with HADS-D scores; only ADI-12-total and -ME scores were significantly related to HADS-A (Table 3).

**Table 3:** Correlation coefficients between ADI-12 and psycho-social measures.

	**HADS-A**	**HADS-D**	**MQoL total**	**MQoL physical**	**MQoL psychological**	**MQoL existential^†^**	**MQoL support^†^**
ADI-12 total	0.35^**^	0.54^**^	−0.54^**^	−0.43^**^	−0.53^**^	−0.41^**^	−0.09
ADI-12-ME	0.42^**^	0.53^**^	−0.59^**^	−0.42^**^	−0.57^**^	−0.46^**^	−0.15
ADI-12-A	0.18	0.42^**^	−0.34^**^	−0.33^**^	−0.35^**^	−0.26^*^	−0.01

*ADI-12, ALS Depression Inventory-12; ME, Negative Mood and Lack of Energy component of ADI-12; A, Anhedonia component of ADI-12; HADS-A/-D, Hospital Anxiety and Depression Scale-Anxiety/-Depression; MQoL, McGill Quality of Life Questionnaire*.

† *Spearman's coefficients;*

* *significant at α = 0.05;*

** *significant at α = 0.01*.

All ADI-12 measures proved to be inversely associated with MQoL-total and sub-scale scores, with the exception of MQoL-Support (Table 3).

**Figure 1:**
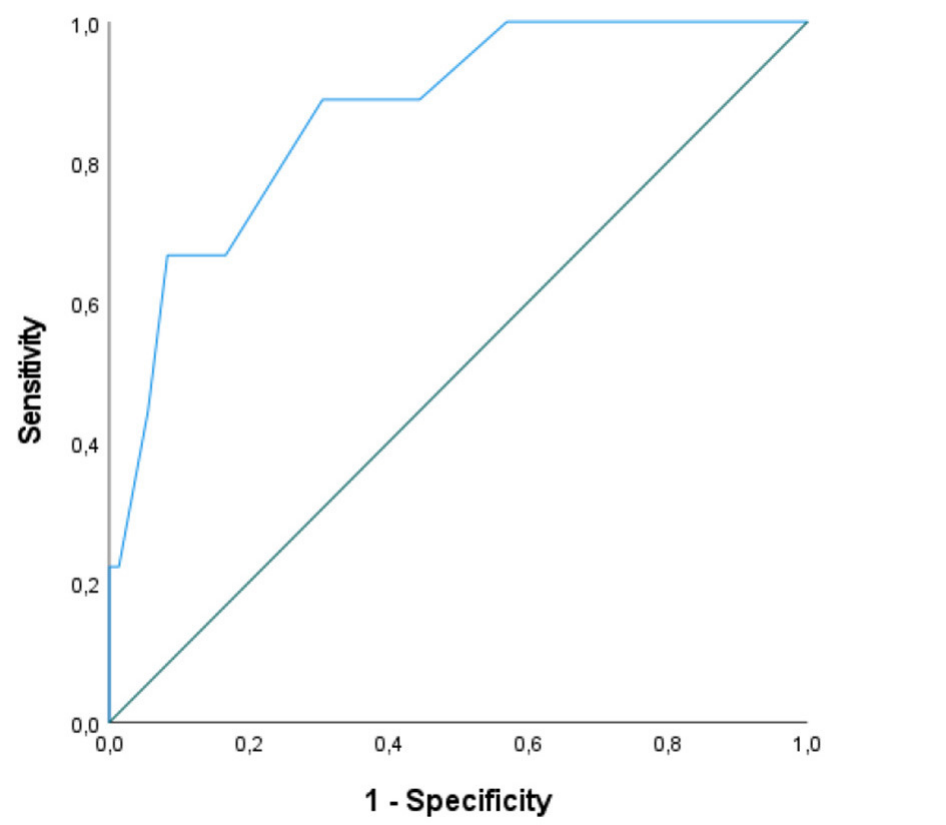
ROC curve for ADI-12 scores as tested against the HADS-D. ROC, receiver-operating characteristic; ADI-12, ALS Depression Inventory-12; HADS-D, Hospital Anxiety and Depression Scale-Depression. A HADS-D ≥ 10 was addressed as indexing depression. 88.9% of sensitivity and 69.4% of specificity at a cut-off of 27.5 (AUC = 0.87; SE = 0.06; 95% CI [0.75, 0.98]).

By regarding an HADS-D score ≥10 as suggestive of depression (15), an optimal cut-off (88.9% of sensitivity and 69.4% of specificity; *J* = 0.58) for the ADI-12 as a whole (i.e., the sum of ADI-A and ADI-ME items) was set at 27.5 (AUC = 0.87; *SE* = 0.06; 95% CI [0.75,0.98]) (Figure 1). When addressing a cut-off of 28, 35.3% of the present patients were classified as having depressive symptoms—whereas, by contrast, HADS-D detected depressed mood in 11.1% of the cohort.

ADI-12 measures proved not to be related with MMSE scores. No association was detected between ADI-12 measures and either disease duration, diagnostic delay, clinical phenotype or site of onset.

## Discussion

The present study provides clinicians with evidence regarding the psychometric goodness and feasibility of the ADI-12 for detecting depressive symptoms in ALS patients. The ADI-12 indeed showed high internal consistency as well as both construct and convergent validity. According to the present results, a cut-off of 28 is proposed for normal range.

The discrepancies reported here in detection rates between HADS-D (11.1%) and the ADI-12 (35.3%) support the notion that ALS-specific, motor-free tools are needed when screening for depression in this population. In this regards, motor-related HADS-D items were actively discussed with patients during administration procedures—i.e., potential biases were to an extent controlled for.

The construct underlying the ADI-12 has been originally identified as mono-factorial—encompassing depressed mood, lack of energy and anhedonia (9). Conversely, the present work hints at anhedonia being separable from depressed mood and asthenia in this population.

The lack of association between ADI-12 scores and the MMSE suggests that depression levels as assessed by the former might be to an extent independent from FT spectrum disorders (3). Thereupon, the ADI-12 is likely to help clinicians differentiating psychogenic/reactive depression from behavioral, apathy-related symptoms in this population (20).

Moreover, the present findings do not support the previously-endorsed notion of longer diagnostic delay and bulbar onset/involvement as risk factors for depression in ALS patients (21, 22). These inconsistencies are likely to be due to the fact that, within studies reporting such findings, psychometric measures of depression were not motor-free (21, 22).

Finally, data here presented again support the interplay between mood and quality of life in ALS patients (23). More specifically, depression levels were strongly associated with a decrease of both physical and psychological well-being but not to the degree of perceived social support, contrarily to previous findings (24). This latter discrepancy might be again linked to the adoption of non-ALS-specific tools for assessing depressive symptoms (24).

Future investigations may focus on: (1) exploring the interplay between the ADI-12 and ALS-specific cognitive screeners (25–29); (2) testing the divergent validity of the ADI-12 against ALS-specific/motor-free scales of apathy (30, 31); (3) investigating the association between the ADI-12 and tools designed to detect FT-like behavioral changes (32, 33); (4) further examining how ADI-12 scores relate motor-functional impairment in ALS patients (34).

In conclusion, the adoption of the ADI-12 as a motor- and FT impairment-independent tool to screen depression in ALS patients should be encouraged in clinical practice.

## Data Availability Statement

The raw data supporting the conclusions of this article will be made available by the authors, without undue reservation.

## Ethics Statement

The studies involving human participants were reviewed and approved by Ethics Committee of Istituti Clinici Scientifici Maugeri, Pavia, Italy. The patients/participants provided their written informed consent to participate in this study.

## Author Contributions

MM, MG, and EA contributed to methodology and statistics. DP contributed to study conception and design, data collection and methodology and statistics. GM supervised the whole process. All the authors contributed to manuscript drafting.

## Conflict of Interest

The authors declare that the research was conducted in the absence of any commercial or financial relationships that could be construed as a potential conflict of interest.

## Publisher's Note

All claims expressed in this article are solely those of the authors and do not necessarily represent those of their affiliated organizations, or those of the publisher, the editors and the reviewers. Any product that may be evaluated in this article, or claim that may be made by its manufacturer, is not guaranteed or endorsed by the publisher.

## References

[1] Atassi N Cook A Pineda CM Yerramilli-Rao P Pulley D Cudkowicz M. Depression in amyotrophic lateral sclerosis. Amyotroph Lateral Scler. (2011) 12:109–12. 10.3109/17482968.2010.536839 21091399 PMC3155886

[2] Roos E Mariosa D Ingre C Lundholm C Wirdefeldt K Roos PM. Depression in amyotrophic lateral sclerosis. Neurology. (2016) 86:2271–7. 10.1212/WNL.0000000000002671 27164661 PMC4909561

[3] Zucchi E Ticozzi N Mandrioli J. Psychiatric symptoms in amyotrophic lateral sclerosis: beyond a motor neuron disorder. Front Neurosci. (2019) 13:175. 10.3389/fnins.2019.00175 30914912 PMC6421303

[4] Strong MJ Abrahams S Goldstein LH Woolley S Mclaughlin P Snowden J. Amyotrophic lateral sclerosis-frontotemporal spectrum disorder (ALS-FTSD): revised diagnostic criteria. Amyotroph Lateral Scler Frontotemporal Degener. (2017) 18:153–74. 10.1080/21678421.2016.1267768 28054827 PMC7409990

[5] Chiò A Logroscino G Hardiman O Swingler R Mitchell D Beghi E. Prognostic factors in ALS: a critical review. Amyotroph Lateral Scler. (2009) 10:310–23. 10.3109/17482960802566824 19922118 PMC3515205

[6] Ferentinos P Paparrigopoulos T Rentzos M Zouvelou V Alexakis T Evdokimidis I. Prevalence of major depression in ALS: comparison of a semi-structured interview and four self-report measures. Amyotroph Lateral Scler. (2011) 12:297–302. 10.3109/17482968.2011.556744 21428731

[7] Heidari ME Nadali J Parouhan A Azarafraz M Irvani SSN Gharebaghi A. Prevalence of depressive disorder among amyotrophic lateral sclerosis (ALS) patients: systematic review and meta-analysis. J Affect Disord. (2021) 287:181–90. 10.1016/j.jad.2021.03.015 33799036

[8] Kübler A Winter S Ludolph AC Hautzinger M Birbaumer N. Severity of depressive symptoms and quality of life in patients with amyotrophic lateral sclerosis. Neurorehabil Neural Repair. (2005) 19:182–93. 10.1177/1545968305276583 16093409

[9] Hammer EM Häcker S Hautzinger M Meyer TD Kübler A. Validity of the ALS-Depression-Inventory (ADI-12)—a new screening instrument for depressive disorders in patients with amyotrophic lateral sclerosis. J Affect Disord. (2008) 109:213–9. 10.1016/j.jad.2007.11.012 18262283

[10] Chiò A Calvo A Moglia C Mazzini L Mora G. Phenotypic heterogeneity of amyotrophic lateral sclerosis: a population based study. J Neurol Neurosurg Psychiatr. (2011) 82:740–6. 10.1136/jnnp.2010.235952 21402743

[11] Measso G Cavarzeran F Zappala G Lebowitz BD Crook TH Pirozzolo FJ. The Mini-Mental State Examination: normative study of an Italian random sample. Dev Neuropsychol. (1993) 9:77–85. 10.1080/87565649109540545

[12] Magni E Binetti G BIanchetti A Rozzini R Trabucchi M. Mini-Mental State Examination: a normative study in Italian elderly population. Eur J Neurol. (1996) 3:198–202. 10.1111/j.1468-1331.1996.tb00423.x 21284770

[13] Gosselt IK Nijboer TC Van Es M. An overview of screening instruments for cognition and behavior in patients with ALS: selecting the appropriate tool for clinical practice. Amyotroph Lateral Scler Frontotemporal Degener. (2020) 21:324–36. 10.1080/21678421.2020.1732424 32157912

[14] Sguazzin C Giorgi I Alesii A Fini M. Italian validation of the McGill Quality of Life Questionnaire (MQOL-It). Giornale Italiano di Medicina del Lavoro ed Ergonomia. (2010) 32:B58–62. 21299077

[15] Costantini M Musso M Viterbori P Bonci F Del Mastro L Garrone O. Detecting psychological distress in cancer patients: validity of the Italian version of the Hospital Anxiety and Depression Scale. Support Care Cancer. (1999) 7:121–7. 10.1007/s005200050241 10335929

[16] Gibbons CJ Mills RJ Thornton EW Ealing J Mitchell JD Shaw PJ. Rasch analysis of the hospital anxiety and depression scale (HADS) for use in motor neurone disease. Health Qual Life Outcomes. (2011) 9:1–8. 10.1186/1477-7525-9-82 21955749 PMC3192662

[17] IBM Corp. IBM SPSS Statistics for Windows, Version 27.0. Armonk, NY: IBM Corp (2021).

[18] Kim HY. Statistical notes for clinical researchers: assessing normal distribution (2) using skewness and kurtosis. Restor Dentist Endod. (2013) 38:52–4. 10.5395/rde.2013.38.1.52 23495371 PMC3591587

[19] Fluss R Faraggi D Reiser B. Estimation of the youden index and its associated cutoff point. Biom J. (2005) 47:458–72. 10.1002/bimj.200410135 16161804

[20] Levy ML Cummings JL Fairbanks LA Masterman D Miller BL Craig AH. Apathy is not depression. J Neuropsychiatr Clin Neurosci. (1998) 10:314–9. 10.1176/jnp.10.3.314 9706539

[21] Hillemacher T Gräßel E Tigges S Bleich S Neundörfer B Kornhuber J. Depression and bulbar involvement in amyotrophic lateral sclerosis. Amyotroph Lateral Scler Other Motor Neuron Disord. (2004) 5:245–9. 10.1080/14660820410021294 15799555

[22] Caga J Ramsey E Hogden A Mioshi E Kiernan MC. A longer diagnostic interval is a risk for depression in amyotrophic lateral sclerosis. Palliat Support Care. (2015) 13:1019–24. 10.1017/S1478951514000881 25137152

[23] Pagnini F. Psychological wellbeing and quality of life in amyotrophic lateral sclerosis: a review. Int J Psychol. (2013) 48:194–205. 10.1080/00207594.2012.691977 22731673

[24] Matuz T Birbaumer N Hautzinger M Kübler A. Coping with amyotrophic lateral sclerosis: an integrative view. J Neurol Neurosurg Psychiatr. (2010) 81:893–8. 10.1136/jnnp.2009.201285 20587497

[25] Woolley SC York MK Moore DH Strutt AM Murphy J Schulz PE. Detecting frontotemporal dysfunction in ALS: utility of the ALS Cognitive Behavioral Screen (ALS-CBS™). Amyotroph Lateral Scler. (2010) 11:303–11. 10.3109/17482961003727954 20433413

[26] Abrahams S Newton J Niven E Foley J Bak TH. Screening for cognition and behaviour changes in ALS. Amyotroph Lateral Scler Frontotemporal Degener. (2014) 15:9–14. 10.3109/21678421.2013.805784 23781974

[27] Poletti B Solca F Carelli L Madotto F Lafronza A Faini A. The validation of the Italian Edinburgh cognitive and behavioural ALS screen (ECAS). Amyotroph Lateral Scler Frontotemporal Degener. (2016) 17:489–98. 10.1080/21678421.2016.1183679 27219526

[28] Siciliano M Trojano L Trojsi F Greco R Santoro M Basile G. Edinburgh Cognitive and Behavioural ALS Screen (ECAS)-Italian version: regression based norms and equivalent scores. Neurol Sci. (2017) 38:1059–68. 10.1007/s10072-017-2919-4 28332040

[29] Tremolizzo L Lizio A Santangelo G Diamanti S Lunetta C Gerardi F. ALS Cognitive Behavioral Screen (ALS-CBS): normative values for the Italian population and clinical usability. Neurol Sci. (2020) 41:835–41. 10.1007/s10072-019-04154-1 31807998

[30] Radakovic R Stephenson L Colville S Swingler R Chandran S Abrahams S. Multidimensional apathy in ALS: validation of the Dimensional Apathy Scale. J Neurol Neurosurg Psychiatr. (2016) 87:663–9. 10.1136/jnnp-2015-310772 26203157

[31] Santangelo G Raimo S Siciliano M D'Iorio A Piscopo F Cuoco S. Assessment of apathy independent of physical disability: validation of the Dimensional Apathy Scale in Italian healthy sample. Neurol Sci. (2017) 38:303–9. 10.1007/s10072-016-2766-8 27844173

[32] Elamin M Pinto-Grau M Burke T Bede P Rooney J O'Sullivan M. Identifying behavioural changes in ALS: validation of the Beaumont Behavioural Inventory (BBI). Amyotroph Lateral Scler Frontotemporal Degener. (2017) 18:68–73. 10.1080/21678421.2016.1248976 27894191

[33] Iazzolino B Pain D Laura P Aiello EN Gallucci M Radici A. Italian adaptation of the Beaumont Behavioral Inventory (BBI): psychometric properties and clinical usability. Amyotroph Lateral Scler Frontotemporal Degener. (2021) 1–6. 10.1080/21678421.2021.1946085. [Epub ahead of print]. 34279169

[34] Cedarbaum JM Stambler N Malta E Fuller C Hilt D Thurmond B. The ALSFRS-R: a revised ALS functional rating scale that incorporates assessments of respiratory function. J Neurol Sci. (1999) 169:13–21. 10.1016/S0022-510X(99)00210-5 10540002

